# Bioactives from Agri-Food Wastes: Present Insights and Future Challenges

**DOI:** 10.3390/molecules25030510

**Published:** 2020-01-24

**Authors:** Sana Ben-Othman, Ivi Jõudu, Rajeev Bhat

**Affiliations:** 1 ERA Chair for Food (By-) Products Valorisation Technologies of the Estonian University of Life Sciences (VALORTECH), Estonian University of Life Sciences, Fr.R.Kreutzwaldi 56/5, 51006 Tartu, Estonia; sana.benothmanepaloulou@emu.ee (S.B.-O.); ivi.joudu@emu.ee (I.J.); 2 Chair of Food Science and Technology, Institute of Veterinary Medicine and Animal Sciences, Estonian University of Life Science, Fr.R.Kreutzwaldi 56/5, 51006 Tartu, Estonia

**Keywords:** waste valorisation, sustainability, bioactive compounds, phytochemicals, bioactivity

## Abstract

Sustainable utilization of agri-food wastes and by-products for producing value-added products (for cosmetic, pharmaceutical or food industrial applications) provides an opportunity for earning additional income for the dependent industrial sector. Besides, effective valorisation of wastes/by-products can efficiently help in reducing environmental stress by decreasing unwarranted pollution. The major focus of this review is to provide comprehensive information on valorisation of agri-food wastes and by-products with focus laid on bioactive compounds and bioactivity. The review covers the bioactives identified from wastes and by-products of plants (fruits, exotic fruits, vegetables and seeds), animals (dairy and meat) and marine (fish, shellfish seaweeds) resources. Further, insights on the present status and future challenges of sustainably utilizing agri-food wastes/by-products for value addition will be highlighted.

## 1. Introduction

Environmental stress created by agriculture-based food wastes and by-products are enormous. In today’s global scenario, sustainable utilization of agri-food waste and/or by-products to produce value-added products for potential applications in cosmetic, pharmaceutical or food industrial uses can provide considerable opportunities for earning additional income for the dependent industry. Besides, valorisation of agri-food wastes and by-products can ensure regional food security and thereby assure sustainable food production [1]. Globally, massive amounts of agri-food wastes and by-products are generated in the agri-food industrial sector. These can occur both at the ‘on farm’ and ‘off farm’ levels. Agricultural wastes (almost reaching up to 50%) not only create safe disposal issues, but also contributes to negative environmental impacts. As per the Food and Agriculture Organization of the United Nations (FAO) report, vegetable wastes have created a significantly higher ‘carbon footprint’ while fruit wastage occurs as the major ‘blue water hotspot’, especially in the industrialized countries covering Europe and Asia [2]. Further, FAO has estimated that globally one third of all the food produced is either wasted or lost, among which the major share goes to fruit, vegetables, and seafood industry. Annually, on a global scale, total value of food lost or wasted is estimated to be US$1 trillion [3]. Also, as per the FAO, to achieve and to ensure the success of ‘Sustainable Development Goals’ it is important that appropriate steps are taken to minimize the wastes generated in the agri-food sector [3].

**Figure 1:**
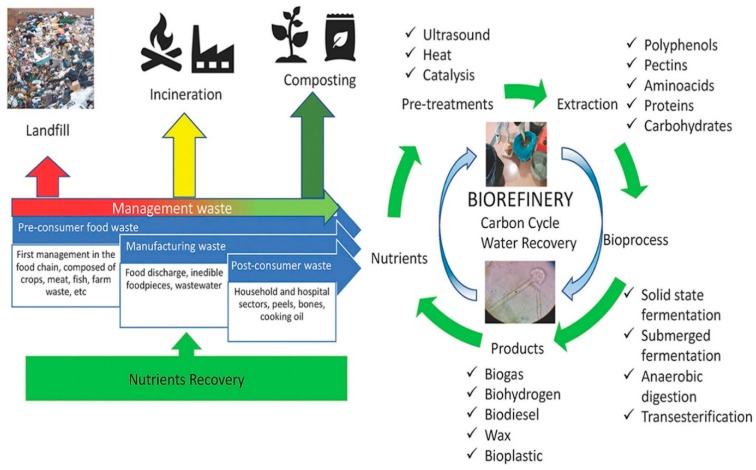
Graphical extract indicating valorisation of food waste based on the concept of biorefinery (reproduced from [7] with permission from Elsevier License number; 4681870689768; dt. Oct 04, 2019)

**Figure 2:**
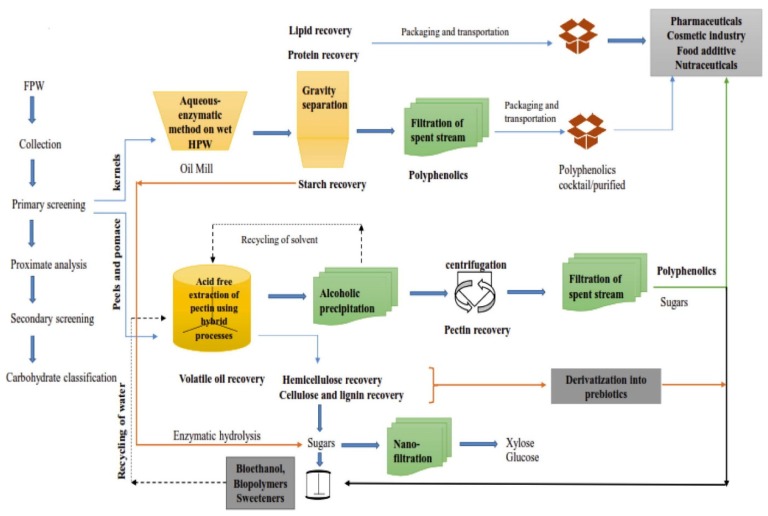
An integrated biorefinery model for fruit processing waste (FPW). As per the authors, this model is based on ‘fractionation strategy’ to improve the cost-efficiency of FPW valorization. Recovery of lipids from fruit kernels can be followed by extraction of proteins and polyphenols. Peels and pomace can be used for the recovery of soluble dietary fibers like pectin and polyphenols in one step extraction followed by alcoholic precipitation (reproduced from [8] with permission from Elsevier License number 4681860844753; dt. Oct 04, 2019).

To date, most agri-food wastes have been utilized as a source of fuel or livestock feeds or as organic fertilizers. Today, with the availability of modern day technologies along with ‘Green Chemistry’ principles, new concepts have been established leading to effective utilization of wastes and by-products of the agri-food sector towards producing value-added products. For instance, advanced spectroscopic techniques such as Fourier Transform Infrared (FTIR) spectroscopy is an valuable tool that can be used for the analysis of functional qualities of different products obtained from food waste like fatty acids methyl esters and glycerin [4,5]. Moreover, eutectic solvents represent a novel form of ‘green solvent’ produced via natural and renewable materials like that of glycerol and salts of organic acids. These eutectic mixtures can be effectively used for the extraction of bioactive compounds such as polyphenols from food industry by-products. Besides, these solvents have been proposed to be an efficient, non-toxic and low cost alternative to organic solvents [6]. Some of the value-added products includes bioethanol, organic acids, enzymes, bioactive functional phytonutrients, prebiotics, etc. In Figure 1 and Figure 2, valorisation of food waste based on its composition through the concept of bio-refinery as well as an integrated biorefinery model for fruit processing waste is shown, which is rather self-explanatory [7,8]. Likewise, to edible portions of an agri-food produce, non-edible portions, which may be in the form of by-products or residues can also encompass high amounts of phytonutrients or nutraceutically valued bioactive compounds exhibiting a wide range of bioactivities. Bioactive compounds isolated from fruits and vegetables wastes or by-products mainly include polyphenols, tannins, flavonoids, flavanols, vitamins (A and E), essential minerals, fatty acids, volatiles, anthocyanins and pigments, whereas, animal-based ones include bioactive peptides and in the dairy industry it is mainly those of whey and colostrum. In the marine sector, the review focuses on the bioactives obtained via fish and shellfish processing industry as well as those of seaweeds.

Nevertheless, valorisation of wastes and by-products can contribute to minimal waste generation or fulfil the widely popular ‘zero waste concept’ to meet the present day needs and demands of the consumer and society. In the present review, we have aimed towards comprehensively collating some of the vital information’s published on wastes and by-products incurred in the agri-food sector, and to the authors knowledge this is the first comprehensive review detailing the potentiality of tapping bioactive compounds from wastes and/or by-products in the entire agriculture based food sector.

## 2. Bioactive Compounds from Fruit Processing Wastes and By-Products

From the available research data, it is evident that much of the wastes and by-products of fruit industry arises after pressing the juice or after producing value-added products. Non-edible parts of fruits such as peels or skin portion and twigs often contain higher amounts of bioactive compounds when compared to the edible parts [9,10]. For example, peels of apple, grapes, citrus fruits and seeds of jackfruit, avocado and mango, are reported to have more than 15% higher content of polyphenolic compounds than pulp [9,11].

Besides, fruits after the production of beverages (in the food industry) generates huge volume of wastes, which are in the form of pomace (a mixture of pulp, skin, seeds, and stem). Owing to high perishability of the pomace, severe technical and environmental problems are incurred [12]. To cope with this problem, it is recommended to use fruit pomace and other fruit processing wastes as livestock feed, or transformed them into bio-fertilizers via composting as well as a potential source of biomass in the production of biofuels [8]. In most instances, fruit processing waste occurring as pomace contains much higher amounts of valuable bioactive compounds than the fruit juice itself [12]. Hence, fruit pomace occurring in larger volumes can represent an interesting natural bio-resource, owed to their chemical richness and heterogeneity [13,14,15]. A wide array of studies have been conducted on valorisation of fruit processing wastes into value-added products. Fruit pomace like those of apple and berries have been proposed as additive in the formulation of bakery and dairy products to enhance their contents in natural antioxidants and dietary fibres. In addition, the presence of natural pigments and volatile compounds can ameliorate the sensory quality of the final product [12].

Further, in Table 1 and Table 2, we have summarized some of the important fruit processing wastes, bioactive compounds isolated and their potential functions, especially those of popular and exotic fruits. In the preceding text, some examples of wastes generated via processing of fruits at the food industrial levels are discussed.

### 2.1. Popular Fruits

#### 2.1.1. Apple

Apple (*Malus domestica* Borkh.) is a widely consumed and well admired fruit for its pleasant taste and aroma as well as for the proven health benefits. The global production of apple exceeded 83 million tons (in 2017) as per the FAO statistics [80]. A major portion of apple production is either consumed raw or converted into value-added products like processed juice or cider which results in the production of huge volumes (~ 25% of the fresh fruit weight) of pomace as a by-product [17]. In addition to its traditional use as animal feed and fertilizer, apple pomace forms an important source of pectin (nearly 14% of the world’s pectin production is extracted from apple pomace). Apple pectin finds wide applications in food, cosmetics and pharmaceutical industries as a thickener, gelling agent, and/or as a food stabilizer. Since the early 2000s, the role of pectin as dietary fibre and prebiotic is well established. Moreover, pectin is recognized to be a good source of nutritional supplement, which contributes towards reduction in blood cholesterol level, post-prandial glycaemic response as well as enhancing satiety [81]. Recently, Wang et al. [16] proposed a new enzymatic process to produce pectin oligosaccharides from apple pomace, which can have better prebiotic properties than pectin. Apple pomace contains ample amounts of health promoting phytochemicals, including those of phenolic acids, flavanols, flavonols, anthocyanins, and dihydrochalcones [18,19]. The major components of pomace such as phenolics are recognised for their potential radical scavenging activity, ability to inhibit protein glycation and anti-tumor activities. Apple-derived by-products contain significant amounts of phlorizin, which is well-established for its role as an anti-diabetic agent. This phlorizin is capable of inhibiting glucose transport effectively via binding of glucose moiety to Na^+^/glucose co-transporter SGLT2 [19,82]. In a recent study by Antika et al., [22] in cell-based and aged mouse models, the potential of dietary phlorizin and phloretin as a therapeutic agent for inhibiting senile osteoporosis has been ascertained.

#### 2.1.2. Banana

Banana (*Musa* L. sp.) is a widely consumed popular tropical fruit with over 113 million tons produced in 2017 [80]. The peel, which forms a part of the non-edible portion, (accounting for ~ 35% of the whole fruit weight), is discarded as a waste. Peel has been traditionally used as a remedy for treating common ailments like cough, burns and inflammation, as well as for managing anaemia and diabetes [83,84]. Banana peel is considered to be a promising raw material source for the isolation of nutraceuticals related to its healing properties. Banana peel is a good source of dietary fibre, potassium, polyphenolic compounds, and essential amino acids. Polyphenolic compounds in peel are three times much higher in concentration than fruit flesh [85]. Phenolic acids, flavonols, flavanols, and catecholamines have been isolated from banana peel [83,86].

With regard to bioactivity, banana peel extract is reported to exhibit strong antioxidant, anti-bacterial, and anti-fungal activities, in addition to providing other health benefits like reducing blood sugar, lowering cholesterol, anti-angiogenic activity, neuroprotective effect, and others [40]. Further, Vu et al. [84] reported ripening stages and processes to impart significant effects on polyphenolic composition and antioxidant capacity of banana peel extracts. Antioxidant capacity of peel extracts was linked with banana ripening stages wherein the activity increased in ripe fruits while it decreased in overripe fruits. Gurumallesh et al. [87] isolated a novel metalloprotease from banana peel which had high potential to be used as a therapeutic for anti-cancer activity (its mechanism involves breaking down of collagen peptide bonds).

#### 2.1.3. Berries 

Different types of berries have been consumed since time immemorial for their rich nutraceutical values. Fruit berries are either consumed fresh, frozen or as processed value added products like juice, jam, etc. Owing to the positive effects imparted, berries and their extracts are gaining much importance in the health and food sector. They are used as an added ingredient in dietary supplements and in functional food formulations [88]. Berry press residues, obtained after juice extraction, are excellent source of phenolic compounds. Klavins et al. [41] reported that berries press residues from *Vaccinum* L. genus berries (bilberries, blueberries, lingonberries, cranberries) to be an excellent source of anthocyanins which are helpful for the prevention of various chronic diseases such as artherosclerosis, cancer and cardiovascular disease. Kitrytė et al. [89] via use of enzyme-assisted extraction recovered phenolic compounds from chokeberry press residues. This extracts contained mainly phenolic acids and flavonols which are well established for their bioactivities. Another interesting source of bioactive compounds from berries processing waste is the branches from berries that grow in clusters like elderberry. Silva et al. [42] reported the potential use of branches obtained from elderberry processing waste to recover high-value nutraceuticals like anthocyanins.

#### 2.1.4. Citrus fruits

Citrus fruits (*Rutaceae* Juss. family) production exceeded 132.9 million tons in 2017 on a global scale. Oranges are the most produced, consumed and processed citrus fruits (73.3 million tons produced in 2017) followed by tangerines, mandarins, and clementines (33.4 million tons), lemons and limes (17.2 million tons), grapefruits and pomelo (9 million tons) [80]. Nearly 40–50% of citrus fruit production is destined for industrial processing, mainly juice, jam, and marmalade. Citrus fruits processing generates huge amount of waste ranging approximately 50–70% of the wet weight of the processed fruit (this depends on the cultivar and processing technology used) [90]. The processing waste generated is traditionally used as animal feed or directly discarded as a waste without further treatments leading to serious environmental problems. Besides, owing to strong anti-microbial activity (owed to essential oils), there might be issues related to inhibition of natural soil microflora [91].

Considering the economic and environmental burden, studies have been conducted on valorisation of citrus processing wastes. One of the most important uses of citrus peel waste is the production of pectin. Almost 85% of pectin production originates from citrus peels (56% from lemons, 30% from limes, and 13% from oranges). Pectin obtained from citrus peels (citrus pectin) is appreciated for its functional properties and is routinely used as gelling agent, food thickener and stabilizer. Besides, it finds wide applications in cosmetic and pharmaceutical industries too [81]. Several studies have also showed the importance of citrus pectin as a nutraceutically valued compound. Citrus pectin has a beneficial role as a dietary fibre imparting prebiotic effects as well as has a positive role in cholesterol metabolism, lowering of blood pressure and controlling of blood glucose [24,25]. Several studies have also reported that citrus pectin directly affects immune cells to regulate inflammatory responses. Citrus pectin is linked with alleviation in the endotoxin-induced pro-inflammatory responses, shown via in vitro and in vivo studies [26,92].

Modified citrus pectin (MCP) is obtained by chemical (acid or alkali treatment), enzymatic or thermal modification of commercial citrus pectin generating oligomers of polygalacturonic acid and rhamnogalacturonan (RGI) regions. In the United States, MCP is registered as a dietary supplement. In addition, several clinical trials conducted have confirmed its potential as mammalian anti-cancer agent [27]. MCP’s anti-cancer effect is mediated by specific molecular interactions with galectin-3, a β-galactoside-binding lectin with varied biological functions. Extracellular galectin-3 is reported to play a vital role in tumour progression and metastasis [93,94].

Citrus fruits processing wastes are also a valuable source of phytochemicals. The phenolic compounds from citrus wastes have antioxidant, anti-inflammatory, and anti-cancer properties, demonstrated via in vitro and in vivo studies [28]. In addition to phenolic acids and flavonoids, citrus wastes especially the seeds contain limonoids a unique class of bioactive compounds [95]. Among these, limonin, a triterpenoid possesses anti-inflammatory, anti-cancer, anti-bacterial, and antioxidant activities [31]. Russo et al. [29,30] analysed samples of lemon and orange juice, seeds, peel, and pulp derived from the industrial transformation process. Results of these studies confirmed that all by-products contain variety of phytochemicals with potential role as nutraceuticals. Lemon peel and pulp had flavones (apigenin-glucoside and diosmetin-glucoside), flavanones (eriocitrin and hesperidin), and a relatively lower amount of limonoids (ichangin). While orange solid waste (pulp and peel) contained high amounts of phenolic acids (hydroxybenzoic and caffeic acids) and flavanones (hesperidin and narirutin).

#### 2.1.5. Mango

Over 50.6 million tons of mango (*Mangifera indica* L.), a popular tropical fruit crop, were estimated to be produced in 2017 [80]. Mango fruits are mainly consumed fresh or used for cooking, but are also canned, frozen, mashed, dehydrated, or prepared as juice or jam [35]. The industrial processing of mango fruit generates about 40–60% of waste: 12–15% of peels and 15–20% of kernel seeds [36]. The mango kernel is a promising source of nutraceuticals and is characterised by its high content of phytochemicals such as phenolic acids, flavonoids, catechins, hydrolysable tannins, and xanthanoids [35,36]. Mangiferin, an important bioactive compound isolated from mango seed and peel shows strong antioxidant capacity and exhibits anti-tumour, anti-bacterial, anti-viral, and immunomodulatory effects [37]. Mango peel contains significant amounts of dietary fibre (45–78%), phenolic acids, flavonoids, xanthones, carotenoids, vitamin C and tocopherol [38,96].

#### 2.1.6. Plum

Plums (*Prunus domestica* L.) global production extended up to 11.7 million tons in 2017 [80]. Plums are widely used for the production of dried fruits, jams, and juices. During their processing, fruits are first pitted, generating an important amount of plum stones consisting of a hull covering a seed inside. Plum pomace, a mixture of peel and pulp is also produced after juice extraction [34,97]. Plum pomace is an important source of phenolic acids, flavonols and anthocyanins, which are all well established for their bioactivities as antioxidants and antimicrobial compounds [32]. Dulf et al. [33] reported that solid state fermentation with filamentous fungi such as (*Aspergillus niger* and *Rhizopus oligosporus*) of plum pomace enhance the extraction yield of total phenolic compounds and flavonoids. The same fermentation of plum seeds resulted in an enhanced oil extraction yield and ameliorated the lipids quality attributes by increasing the content of sterol esters and n-3 polyunsaturated fatty acids (PUFA).

### 2.2. Exotic Fruits

The biological meaning of exotic fruit refers to those fruits that are not native to a given area/region/country. These fruits are either intentionally transplanted from another region (non-native) or introduced purposely or accidentally. In the preceding section, we have shortlisted some of the interesting research works that focuses on some of the selected fruits which remains as unconventional fruits and in certain case the reporting researchers consider them as exotic. 

A wealth of traditional knowledge and scientific database are available on the potential health benefits of consuming exotic fruits. Just as an example: in miracle fruit, bioactive compound ‘miraculin’ is identified to impart artificial sweetening effects and can be used by people suffering from diabetes [62]. Further, there are ‘kiwano’ and ‘aguaje’ fruits, which are a good source of vital minerals like potassium and magnesium and vitamins (vitamin A and C). Genovese et al. [98] characterized exotic fruits from Brazil and found ‘Coquinho’ and ‘Camu-camu’ (*Butia capitata* Becc. and *Myrciaria dubia* (Kunth) McVaugh) to have high levels of vitamin C (39.7 and 43%, respectively). Exotic fruit like that of durian is reported to have high amounts of bioactive compounds like polyphenols, flavonoids, anthocyanins, carotenoids, etc [56]. High levels of anthocyanin, quercetin glycoside and carotenoids in exotic fruits like hog plum (*Spondia dulcis* L.), peanut butter fruit (*Bunchosia armeniaca* (Cav.) DC.), chupa-chupa (*Martisia cordata* Humb. & Bompl.) and kwai muk (*Artocarpus hypargyreus* Hance ex Benth.) grown in North Queensland are reported [99]. Colombian cultivar of *Physalis peruviana* L. (an exotic fruit) oil is reported to be a rich source of essential fatty acids [100]. In Table 2, a list of exotic fruits, their botanical classification and countries encountered is provided. However, scientific literature are scarce on effective waste utilization and finding potential nutraceutical applications on most exotic fruits.

Fruits and by-products like that of peel, seeds and leaves of exotic Brazilian fruit Araticum (*Annona crassiflora*) is reported to be rich in bioactive compounds such as alkaloids, annonaceous acetogenins, phytosterols, polyphenolic compounds, carotenoids, tocols, dietary fiber essential minerals, vitamins and oil. These compounds are reported to contribute towards a range of bioactivity like that of anti-inflammatory, antitumor, antidiabetic, antioxidant, anti-diarrhoeic, antimicrobial, anti-parasitic and hepatoprotective activities [46]. Durian skin waste is reported to exhibit high therapeutic value, owed to higher amounts of bioactive compounds benefits such as: possessing anti-microbial, anti-proliferative, anti-hyperlipidemic and anti-diabetic activities [56]. Devalaraja et al. [101] reported for the presence of bioactive proanthocyanidin isolated from persimmon (*Diospyros kaki* L.) fruit peel which exhibited anti-obesity and anti-diabetic effects. Xanthones (α- and β-mangostin) isolated from the skin of mangosteen fruits are well established for their anti-cancer, anti-microbial and anti-cholesterol activities. The peels of exotic mango cultivars (chonsa and langsra) is reported to have high polyphenolic and flavonoid contents [102]. Moriwaki et al. [103] have reported procyanidin extracted from litchi pericarp to be effective in treating hyperuricemia and gout. Rambutan, another exotic fruit of Southeast Asia has been evaluated for bioactive contents in the peel and skin. Accordingly, dried peel had high amounts of vitamin C, dietary fibre and polyphenols (tannins, flavonoids) and polyphenolic acids such as caffeic, coumaric, gallic, syringic, ellagic acids. Industrially valued volatile flavouring compounds such as trans-isoeugenol and eugenol have been isolated from the peel of ripened exotic fruit *Strychnos spinosa* Lam. [104]. It is clear from the available database that waste portions of exotic/unconventional fruits contain rich amounts of bioactive compounds and research undertaken on this is rather scarce in the introduced region, a gap that is expected to be filled in the near future.

## 3. Bioactive Compounds from Vegetable Processing Wastes and By-Products

Vegetables are an important source of phytonutrients that possess health promoting and disease preventive properties. By-products and wastes generated mainly from the inedible parts of the vegetables constitute a valuable source of these phytonutrients and remains under valorised. Vegetables wastes are usually generated at the on farm (during the harvesting) or post-harvesting stages. This includes, left over harvest, inedible parts like leaves, twigs or stems. Popular vegetables like potato, tomato and carrot have long been used in food industry (to produce processed products (like juice, canning, etc), generating enormous quantities of wastes. These wastes generated have extensively studied for their potential usage as natural compost or livestock feed and much more. The emerging trends of ready to eat salads and meals, pre-cut and canned vegetables in the past decade has also led to generation of wastes and by-products which can be valorised [105]. Table 3 summarizes examples of vegetable processing wastes, the isolated bioactive compounds, and their potential health benefits. Some of the popular vegetables wastes, their by-products and bioactives present is discussed in the following section.

### 3.1. Vegetable Sources

#### 3.1.1. Potato

Globally, potato (*Solanum tuberosum* L.) is the fourth main crop produced after rice, wheat and corn, with over 388 million tons produced in 2017 [80]. This tuber crop plays an important role in human diet as a staple food in most households. Large-scale peeling of potatoes for the production of fries, chips, and other potato-based snacks generate huge quantities of peel wastes, which are generally used for the production of biofuels or organic biofertilizers. Potato peel is reported to be a valuable source of bioactive compounds, mainly phenolic acids and glycoalkaloids [108,118]. In fact, potato peel contains much higher amount of phenolic compounds than the flesh. Phenolic acids in potato peel are well established for their antioxidant and antibacterial activities [107]. Moreover, intake of chlorogenic acid, the major phenolic acid extracted from potato peel, has been associated with decreased risk of cardiovascular disease and type 2 diabetes [119]. Glycoalkaloids from potato peel are also gaining importance owed to their anti-carcinogenic properties via induction of cytotoxicity and apoptosis in different cancer cell lines [120]. Potato starch extraction residue can also be explored as a good source of pectin. Oguta and Mu [106] extracted pectin from sweet potato residues which also exhibited good antioxidant activity.

#### 3.1.2. Carrot

Carrot (*Daucus carota* L.) is a widely consumed vegetable and is a rich source of dietary fibres, phenolic compounds, carotenoids, vitamins and essential minerals [110]. In food industry, carrots are used for the production of juice, jams, and in the preparation of ready to eat salads generating waste in the form of peels that accounts for around 11% of the initial weight. This industrial waste is a valuable source of carotenoids and can be sustainably extracted using green extraction techniques like supercritical CO_2_ extraction [109]. In addition, about 25–35% of carrot harvest are discarded owed to irregular size, form or colour. This is generally used as animal feed or even thrown as waste. Discarded carrots can also be used for the extraction of value-added bioactives. Idrovo Encalada et al. [110,121] recently reported that pectin-enriched fraction is obtained using high-power ultrasound extraction with high antioxidant capacity associated to the presence of α- and β-carotenes, lutein, and tocopherols.

#### 3.1.3. Beetroot

Beetroot (*Beta vulgaris* L.) is another widely consumed root vegetable rich in nitrates, flavonoids, carotenoids, betalains, vitamins and minerals [122]. Betalains are water-soluble nitrogen-containing pigments including betacyanins (violet to red colour) and betaxanthins (orang to yellow colour). Betalains is established to be good antioxidant possessing anti-inflammatory, anti-carcinogenic, and anti-microbial properties [123].

Beetroots are used for the preparation of processed foods such as juice, pickles, and prepared meals. The generated waste in form of peels and pomace can be valorised to recover the high-value nutraceuticals or bioactive compounds. Vulić et al. [111] reported beetroot pomace extract to contain phenolic acids, flavonoids and betalains, which exhibited good antioxidant activity (in vitro) and hepatoprotective effects (in vivo). The aerial parts of the beetroot, comprising of leaves and stems are generally discarded after harvest or before processing of the root. Unlike peel and pomace, less attention was placed on this waste despite of its high potentiality. Recently, Lasta et al. [112] reported that extracts from beetroot aerial parts exhibit high antioxidant activities. Further works are warranted to identify the bioactive compounds in these extracts for better utilisation of the waste.

#### 3.1.4. Broccoli and Cauliflower 

Broccoli (*Brassica oleracea* L. *Italica*) is a highly valued vegetable, the consumption of which has increased tremendously over the past few years. Recent reports indicate global production of broccoli and cauliflower to have increased from 8.1 million ton during 1987 to nearly 26 million ton in 2017 [80]. The increased interest in this vegetable is associated with reduced indices of different types of cancers. The chemopreventive effect is mainly attributed to the presence of glucosinolates, sulphur-containing plant secondary metabolites, and their degradation products [113,114].

Generally, the florets representing 10–15% of the total plant mass are consumed, or are used in large-scale preparation of pre-cut and frozen vegetables [124]. The wastes are in the form of leaves and stalk which are usually discarded despite of its similar composition to the florets [114]. Nevertheless, florets that are overripe or have some yellowish spots are also discarded. These residues generated after processing and packaging of broccoli florets can be effectively valorised to obtain bioactive compounds of interest. Thomas et al. [113] highlighted the potential use of broccoli by-products for the extraction of glucosinolates and polyphenolic compounds. Formica-Oliveira et al. [125] reported single or combined UV-B and UV-C irradiation treatments to significantly increase phenolic compounds and glucosinolates contents of broccoli leaves and stalks, thus enhancing its value as a source of bioactives. In addition, broccoli by-products are reported to have high content of proteins (23–25%) and carbohydrates (32–37%) which renders them vital raw material to be used as carrier for stabilizing and delivering bioactive compounds such as epigallocatechin gallate [124].

On the other note, cauliflower (*Brassica oleracea* L. *Botrytis*) is also a popular vegetable belonging to the Brassicaceae family, encompassing higher level of bioactive compounds linked with providing positive health benefits. The non-edible parts: outer leaves, stems and pods that account for about 36% of the total mass are usually discarded as waste. Cauliflower waste extracts were characterized by high content of flavonoid glycosides which were mainly derived from kaempferol and quercetin. Further, sinapic and ferulic acids were the major phenolic acids detected in cauliflower waste extracts [115]. Huynh et al. [126] reported solid state fermentation of cauliflower by-products (via use of filamentous fungi) to significantly enhance the extraction yield of phenolic compounds wherein an improved release of kaempferol glucosides was observed. Kaempferol is a well-studied natural flavonoid imparting anti-inflammatory and anti-carcinogenic properties [127]. Cauliflower waste is also reported to be an important source of isothyiocynates, the product of glucosinolates hydrolysis, which are linked with anti-carcinogenic properties [116]. Further, cauliflower by-products also contains proteins that can be valorised. For instance, Xu et al. [117] isolated bioactive peptides with ACE inhibitory effect from cauliflower leaves protein enzymatic hydrolysate. The authors highlighted that the protein obtained from cauliflower by-products can be a cheap source of functional foods raw material (to treat hypertension related disorders).

#### 3.1.5. Underexplored Vegetable Wastes

Apart from the extensively studied vegetables wastes for recovery of bioactive compounds, there are still a wide group of vegetable wastes that remains underexplored. Some of the wastes includes those generated from mushroom, garlic, eggplant, spinach and other green leafy vegetables, cabbage and other Brassicaceae family, and other traditional vegetables with shorter shelf life. One of the most recently studied material being evaluated is that of onion and garlic skin/peel, which generates huge amount of wastes. The skin portion is reported to be rich in total phenolics, flavonoid, flavonol, quercetin, aglycone, fructans, alk(en)yl cystein sulphoxides and dietary fibre, exhibiting bioactivities like antioxidant, antimicrobial, antispasmodic, and antidiabetic activity [128,129,130]. Similarly, garlic husk has been reported to be a potential source for cellulose (41%), hemicellulose, lignin, and polyphenolic compounds [131].

## 4. Bioactive Compounds from Seeds

Seeds from fruits and vegetables remain underexplored for their potential bioactivity or for presence of nutraceutically-valued bioactive compounds. Many of the literature available indicates the presence of higher amount polyphenolic compounds in fruits seeds (longan, jackfruit, mango, avocados, grapes) when compared to the edible pulp portion. Seeds of avocado and jackfruit are reported to contain high amounts of polyphenols (5160 and 2770 mg/100g, respectively) and carotenoids (630 and 1910 μg/100 g) [132,133,134]. With regard to citrus family, reports are available on lemon and orange seeds. Russo et al. [29] reported lemon seeds to be rich in bioactive phenolic acids (mainly gallic and caffeic acids) and limonoids (ichangin, deacetylnomilin, limonin, nomilin and obacunone). In another study, orange seeds was reported to be an important source of limonoids (limonin, nomilin, obacunone, and ichangin) which also had high content of flavanones (hesperidin and narirutin) [30].

Seeds obtained as a by-product of berry processing is reported to be a valuable source of oil with a unique fatty acid composition occurring in combination with higher content of lipid-soluble antioxidants (mainly tocopherols) [43,44]. Plum (*Prunus domestica*) stones consisting of seed is proposed to be a good source of oil (yielding up to 50% *w*/*w*). Plum seed oil is mainly composed of oleic and linoleic acids with a high ratio of unsaturated/saturated fatty acids (UFA/SFA) which is considered favourable for biodiesel production [97]. However, there is wide gap of research works undertaken on the potential use of this oil for food or pharmaceutical applications. Plum seeds are also reported to be a good source of protein and bioactive peptides. Gonzalez-Garcia et al. [34] proposed enzymatic extraction of bioactive peptides from defatted plum seeds. These bioactive peptides showed antioxidant and angiotensin-converting enzyme (ACE) inhibitory activities that can be related to potential anti-hypertensive capacity.

Mango seeds/kernel which is a major waste after processing holds promise as a potential therapeutic source with numerous bioactive compounds being isolated such as polyphenols, flavonols, alkylresorcinol, xanthones and gallotannins, phytosterols (stigma-sterol, campe-sterol), sito-sterol (b-sito-sterols) and tocopherol [135,136,137]. Further mango kernel/seed is a valuable source of proteins and lipids. Protein extracts from mango seeds is reported to have high essential amino acids index and protein quality index. Mango seeds lipid (5–13%) or the oil has comparable characteristics to that of vegetable butter with high levels of saturated fatty acids (mainly palmitic, oleic and stearic acids), which provides a good stability and a relatively high melting temperature. Hence, mango seed oil can be a potential source to be used in food and cosmetic industries [35,36].

Seeds of *Annona squamosa* or custard apple fruits are poisonous, but they contain acetogenins, which possess phytochemical values as these group of polyketides can be potent inhibitors of mitochondrial complex I, as well as exhibit anti-cancer and pesticidal activities [138]. Further, with regard to pomegranate seed waste (obtained after processing from juice industry), oil extracted is reported to contain high amounts of conjugated fatty acids and dietary fibres [139]. Seeds and seed oil of avocado fruit (*Persea Americana* Mill.) contains high amounts of polyphenols, flavonoids, flavonols, procyanidins, tannins, phenolic acids, hydroxycinnamic acids, and essential fatty acids. Seeds of avocado have been used for treating hypertension, hypercholesterolemia, inflammation and diabetes [140,141]. Seeds of rambutan, an exotic fruit has high amounts of bioactive alkaloids, saponin and tannins [142]. Seeds of the exotic fruit ‘red pitaya’ (*Hylocereus polyrhizus* (F.A.C.Weber) Britton & Rose) is reported to have high amounts of phenolic compound with catechin being the major flavonoid and ascorbic acid content exhibiting good antioxidant activities [143]. Further, grape seed which is one of the much studied raw material is reported to contain bioactive components such as phenols, tannin, resveratrol, quercetin, flavonoids and anthocyanins, exhibiting antimicrobial, antioxidant and anticancer properties along with providing cardiovascular protective effects [144,145,146,147]. Date (*Phoenix dactylifera* L.) seeds wastes are also valued for their bioactive contents such as that polyphenolic compounds, flavonoids, flavonols, anthocyanins, proanthocyanidins and ascorbic acid [148].

On the other hand, there are also the seeds from oil-yielding plants. Rapeseed (*Brassica napus* L.) oil is reported to contain bioactive components such as sterols, tocopherols, polyphenols, flavonoids, tannins and phospholipids which are linked with associated with lowering risks associated with cardiovascular problems, cancer and stroke [145,149].

Camelina (*Camelina sativa* L.) seed oil is reported to be rich in bioactive compounds including those of vital unsaturated fatty acids like omega 3- and -6 fatty acids (linoleic and linolenic acids), phenolic acids, flavonoid aglycons and carotenoids [150]. Further, underutilized legumes and seeds belonging to species of *Canavalia*, *Entada scandens* G.Forst., *Mucuna*, *Nelumbo*, and *Sesbania* is reported to have high amount of bioactive compounds including those of polyphenols, flavonoids, vitamins, etc [151,152,153,154]. Seeds of *Theobroma grandiflorum* (Willd. ex Spreng.) K.Schum. (cupuaçu) is reported to contain bioactive phytochemicals such as sulfated flavonoid glycosides (theograndins I and II), flavonoid antioxidants, catechin, epicatechin, kaempferol, quercetin, quercetin 3-*O*-β-D-glucuronide, isoscutellarein hypolaetin 8-*O*-β-D-glucuronide, and isoscutellarein 8-*O*-β-D-glucuronide 6′′-methyl ester [155].

## 5. Bioactive Compounds from Animal Products Processing Waste

Animal product processing mainly involves the milk and meat processing industries. Both industries generate various by-products that remain significantly undervalorised. In the European Union, animal by-products generated exceeds to 20 million tons annually, originated from slaughterhouses, the meat processing and dairy industries [156]. Disposal and treatment of the generated waste is expensive and can present serious health and environmental problems. Therefore, industries and researchers have focused on converting these by-products into useful sources of value added non-edible products such as fertilizers and biodiesel, and edible products including bioactive compounds like peptides and oligosaccharides. In Table 4, we have summarized examples of bioactive compounds recovered from animal and marine products processing wastes.

### 5.1. Dairy By-Products

Milk is a major source of protein and other nutrients in the human diet and is widely consumed as a drink or in other processed forms. Whey is the major by-product from cheese production, corresponding to the remaining aqueous fraction of milk after casein coagulation. Whey is generated in huge volumes that can cause serious environmental problems, but, it is also recognized as an important source of proteins with excellent nutritional and functional properties which are widely used in various food product development. Whey proteins are also an important source of bioactive peptides obtained by enzymatic hydrolysis exhibiting antioxidant and anti-hypertensive properties [157,173]. Whey protein fraction is recovered as retentate after microfiltration; and the generated permeate is rich in carbohydrates including lactose and other oligosaccharides. This permeate can also be valorised for the isolation of bioactive milk oligosaccharides that have important health benefits such as anti-inflammatory, inhibition of enteric bacteria adhesion to intestinal cells, and promoting *Bifidobacteria* growth [158]. Whey permeate can also be used for the production of galacto-oligosaccharides which can perform the role of prebiotics, by transgalactosylation of lactose using β-galactosidase [159].

Colostrum, the first form of milk produced by mammals immediately after parturition is also much valued for its rich health-promoting effects. The presence of colostrum in the raw milk supply is undesirable due to its sensitivity to heat treatment and the production of off-flavour. Colostrum is an important source of proteins containing 50% of immunoglobulins [174]. Colostrum protein fraction contains lactoferrin, a low molecular weight glycoprotein, with various biological functions including antioxidant, anti-inflammatory, anti-microbial and neuroprotective functions [160]. Colostrum permeate obtained after microfiltration is also an important source of bioactive milk oligosaccharides [161]. As of today, most of the literatures available is on commonly consumed milk from cow, buffalo, sheep and goat, but there is vast scope to explore for other underutilized resources too (camel, donkey, etc).

### 5.2. Meat Processing By-Products

Meat processing industry by-products include blood, bones, horns, skin, fatty tissues, and viscera. The treatment and disposal costs of these wastes can be balanced through innovation to generate value added products. Thus, they have been mainly used for the production of feed and pet food, biodiesel from fats, fertilizers, etc [162]. Meat by-products (meat trimmings, blood, bones and skin collagen) are rich in proteins and can constitute a good substrate for proteolysis. The obtained protein lysate is a valuable source of bioactive peptides with in vitro and in vivo antioxidant, anti-hypertensive, and anti-microbial activities [163]. However, with the available research information, scientific research works undertaken on effective valorisation of wastes and by-products from animal resources remains in infancy stage when compared to fruits or vegetables, and hence detailed research is warranted on this in the near future.

## 6. Bioactive Compounds from Marine Product Processing Wastes

### 6.1. Fish and Shellfish Waste

The fishing industry is a major contributor to the economy of numerous regions and it is opined that this industry contributes to over 170 million tons of annual production as fish or shellfish from fisheries and aquaculture [175]. By-products generated from fishing industry account for 40–50% of the total fish weight. Fishing industry by-products such as anchovy (*Engraulis encrasicolus*), carp (*Cyprinus carpio*), cuttlefish (*Sepia officinalis*), cod (*Gadus morhua*), tuna (*Thunnus albacares*), etc, can be used as animal feed or as a source of value-added minerals such as calcium phosphates [176]. Fish and shellfish by-products are also an important source of bioactive compounds imparting multiple health benefits. For example, several bioactive compounds can be isolated from processing waste of shrimps, one of the most widely consumed seafoods, including chito-oligosaccharides from chitin, astaxanthin a red carotenoid with high antioxidant capacity, and Ω-3 polyunsaturated fatty acids [164,165,166]. Salmon nasal cartilage is a valuable source of bioactive proteoglycans reported for anti-angiogenic activity, relieving joint pain discomfort in elderly people and promoting wound healing [167,168,169,177]. Fish skin is also an important source of collagen that can be hydrolysed to bioactive peptides with multiple health benefits with antioxidant, anti-hypertensive, and anti-diabetic activities, as well as enhancing learning and memory [170,171,172].

### 6.2. Seaweeds

Edible seaweeds has been consumed since time immemorial in China, India, Japan, Korea, and other parts of Southeast Asia. Seaweeds contain rich amount of nutraceutically value phytonutrients and are well established for their role in disease prevention in humans. Antioxidant, anticoagulant, anti-microbial, antidiabetic, anti-obesity, anticancer, anti-inflammatory activities are linked with seaweeds. Most of the edible seaweeds harvest remains underutilized and goes as a waste. Seaweeds belonging to Chlorophyta, Rhodophyta and Phaeophyta are reported to be rich in dietary fibre, sulfated polysaccharides, omega-3-fatty acids, β-carotene, carrageenan, fucoidan, lycopene, polyphenolic compounds, carotenoids gallic acid, quercetin, zeaxanthin, astaxanthin, vitamin C, phlorotannins and phloroglucinol [178,179,180,181]. Available literatures clearly indicate the presence of bioactive compounds in some of the popular and edible seaweeds such as those of *Ulva* spp., *Sargassum polycystum, Caulerpa lentillifera, Kappaphycus alvarezii, Laminaria japonica, Ascophyllum nodosum, Codium* spp., *Gracilaria* spp., *Porphyra umbilicalis, Undaria pinnatifida*, and others. However, identification of bioactive compounds in underutilized seaweeds, especially those harvested from the wild/sea and of those creating seaweed blooms, still remains in infancy stage, a gap that needs to be filled in the near future.

## 7. Conclusions

**Figure 3:**
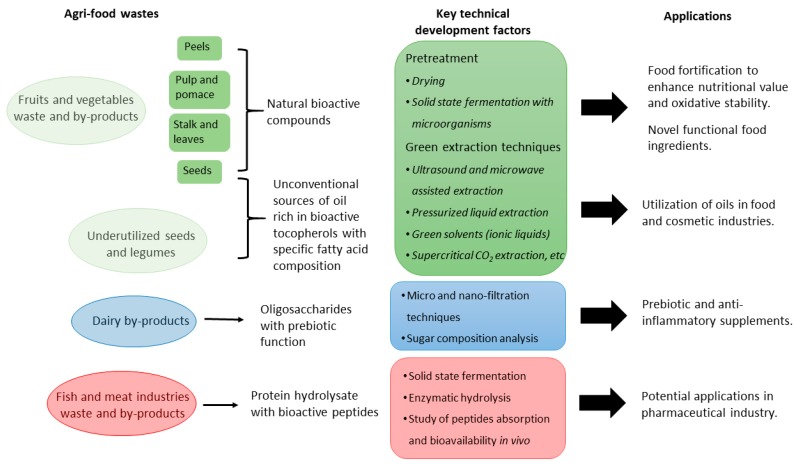
Schematic representation summarizing key technical development factors and potential applications of agri-food wastes and by-products valorisation.

It is evident from the available literature that agri-food wastes and by-products presents wide opportunity for isolation of natural bioactive compounds with possible potential applications in the food, pharma and cosmeceutical industries. In Figure 3, we have provided a schematic representation summarizing key technical development factors and potential applications of agri-food wastes and by-products valorization. 

Most of the research works undertaken is concentrated on industrial wastes obtained post-processing of the raw materials. However, still there are wide gaps and challenges that need to be addressed in relation to underutilized resources like exotic fruits, vegetables, marine and dairy by-products. Apart from isolation and identification of bioactive compounds, it will be worthwhile to explore, evaluate and create a toxicological database on the extracts and understand their potential bioavailability and metabolism. Potential application of the natural bioactives in cosmetic application via support from in vitro and in vivo experiments are essential. Finding the value for isolated natural bioactive compounds, pigments, vitamins, oil and others via fortification in food can open up a new arena in food sector (development of novel functional foods). Fibre extracted from wastes and by-products can find potential applications in food application as a low calorie bulking agent useful as a flour or fat replacer or to improve water and oil absorption and other functional properties and viscosity or as a natural ingredient to provide oxidative stability and enhance the shelf-life of foods. Use of wastes as source of prebiotic oligosaccharides will be an interesting arena to be explored. Oil obtained from underutilized seeds can be explored for their bioactivity as well as can be tried for nano-encapsulation. Besides, husk and oil cake can also be explored for the presence of bioactive compounds and bioactivity. Also, most of the works reported have focused more towards isolation of bioactive compounds from single resources (e.g., apple pomace, grape waste, potato peel waste, etc), thus creating a gap of information from a mixture of raw materials, especially those obtained from food processing industries or those obtained as kitchen wastes in restaurants. Utilizing agri-food wastes and by-products (rich in pectin, fibre, lignin, cellulose and hemi cellulose) for producing novel biodegradable bioplastics is another arena that needs to be investigated. Finally, improving and optimization of the isolation, extraction, processing and production processes of agri-food wastes and by-products via a sustainable approach is the need of the hour.

## Figures and Tables

**Table 1:** Bioactive compounds from popular fruits processing wastes and by-products.

Fruit	Type of Waste	Bioactive Compounds			Bioactivity	Reference
		Class	Concentration (mg/kg *)	Major Compounds		
Apple	Pomace	Carbohydrates	n.a. **	Pectin and pectin oligosaccha-rides	Dietary fibre, prebiotic, Hypo-cholesterolemic	[16]
		Phenolic acids	523–1542	Chlorogenic acid Caffeic acid Ferulic acid *p*-coumaric acid Sinapic acid *p*-coumaroyl-quinic acid	Antioxidant, anti-microbial, anti-inflammatory, anti-tumour, cardio-protective	[17,18,19]
		Flavonoids	2153–3734	Isorhamnetin Kaempferol Quercetin Rhamnetin glycoconju-gates Procyanidin B2 (−)-Epicatechin		
		Anthocyanins	50–130	Cyanidin-3-*O*-galactoside		
		Dihydro-chalcones	688–2535	Phlorizin Phloretein	Anti-diabetic. Potential in treating obesity. Promoting bone-forming blastogenesis.	[20,21,22]
		Triterpenoids	n.a.	Ursolic acid, Oleanolic acid	Anti-microbial, anti-inflammatory	[17,23]
Citrus fruits	Peel	Carbohydrates		Pectin	Dietary fibre, lowering blood pressure, improving blood glucose control, prebiotic effect. Immuno-modulatory.	[24,25,26,27]
				Modified citrus pectin	Anti-cancer agent	
	Peel and pulp	Phenolic acids	276 (Lemon) 560 (Orange)	Hydroxybenzoic acid Caffeic acid	Antioxidant, anti-inflammatory, anti-cancer properties.	[28,29,30]
		Flavones	1659 (Lemon) 55 (Orange)	Apigenin-glucoside Diosmetin-glucoside		
		Flavanones	10646 (Lemon) 22298 (Orange)	Eriocitrin Hesperidin Narirutin		
	Seeds	Limonoids	375 (Lemon) 114 (Orange)	Limonin Nomilin Obacunone Ichangin	Anti-inflammatory, anti-cancer, anti-bacteria, antioxidant activities.	[31]
Plum	Pomace	Phenolic acids	95.7	Neochlorogenic acid Chlorogenic acid	Antioxidants, anti-microbial, prevention of chronic diseases.	[32]
		Flavonols	40.3	Quercetin glycosides Kaempferol Rutinoside		
		Anthocyanins	6.5	Cyanidin glycosides Peonidin glycosides		
	Seeds	Lipids	53% ***	Oil rich in sterol esters and n-3 PUFA		[33]
		Peptides	n.a.	Bioactive peptides from protein hydrolysate	antioxidant activity, ACE inhibitory activity	[34]
Mango	Kernel seed	Phenolic acids	n.a.	Gallic acid and its derivatives	Antioxidant anti-tumour, anti-bacterial, anti-viral, immune-modulatory effect.	[35,36,37]
		Flavonoids	7200–13000	Quercetin Isoquercetin Fisetin		
		Catechins	n.a.	Epicatechin Epigallocatechin Epicatechin gallate		
		Hydrolysable tannins	n.a.			
		Xanthanoids	13600	Mangiferin		
		Carotenoids	7.9			
	Peel	Carotenoids	1900	β-cryptoxanthin Lutein β-carotene	Antioxidant, prevention of age-related macular eye disease, regulation of bone homeostasis.	[38,39]
Banana	Peel	Phenolic acids	99.5	Ferulic acid *p*-Coumaric acid Caffeic acid Sinapic acids	Antioxidant, anti-bacterial, anti-fungal activity, reducing blood sugar, lowering cholesterol, anti-angiogenic activity, neuroprotective effect.	[40]
		Flavonols	1019.6	Rutin, Quercetin Kaempferol Myricitin Laricitrin		
		Catechins	n.a.	Catechin Epicatechin Gallocatechin		
		Catecholamines	4720	Dopamine, L-dopa		
Berries						
*Vaccinum* genus berries (bilberries, blueberries, lingon-berries, cranberries)	Berries press residue	Anthocyanins	284,950 (bilberries) 84,120 (blueberries) 43,530 (cranberries) 27,890 (lingon-berries)	Glycoconjugates of delphinidin cyaniding petunidin malvidin	Prevention of various chronic diseases such as artherosclerosis, cancer, and cardiovascular disease.	[41]
Elderberry	Branche waste	Phenolic acids	45,600	Chlorogenic acid	antioxidant, anti-inflammatory, anti-cancer properties.	[42]
		Flavonols	468,200	Quercetin and its glycoconjugates		
		Anthocyanins	2530	Cyanidin and its glycoconjugates		
Wild and cultivated berries	Seeds	Lipids	14.61–18.19%	Oil rich in α-linoleic acid with a high content of α- and γ-tocopherols	Balancing diet fatty acid composition, Antioxidant, skin regeneration.	[43,44]

* Values are expressed as mg/kg of dry weight. ** n.a. concentration data not available in the literature. *** concentration is expressed as percentage of oil *w*/*w*.

**Table 2:** Bioactive compounds from exotic/ unconventional fruits.

Fruits/English Name	Scientific Name	Bioactive Compounds		Origin/ Countries Encountered	Reference
		Class	Compound		
Aguaje fruit or Moriche palm tree fruit	*Mauritia flexuosa* L.f.	Phenolic compounds Carotenoids Tocopherols Vitamin C Dietary fibre Phytosterols Mono- and poly-unsaturated fatty acids		Native of Peru, Amazon regions of Brazil	[45]
Araticum	*Annona crassiflora* Mart.	Phenolic compounds Alkaloids Annonaceous acetogenins Tocols Carotenoids Phytosterols Dietary fibre Vitamins Minerals Essential oils		Native of Brazil	[46]
Black Sapote or Zapote Blanco or Mamey Sapote	*Diospyros digyna* Jacq.	Polyphenolics Flavonoids Anthocyanins		Native of central Mexico	[47,48]
		Carotenoids	β-carotene Lutein		
		Tocopherols Vitamin C			
Cherimoya or custard apple	*Annona squamosa* L.	Annonaceous Acetogenins Diterpenes Alkaloids Cyclopeptides		Native of South America, but grown in Southern parts of Asia and Europe, and Africa	[49]
Conkerberry or Bush currant	*Carissa spinarum* L.	Coumarin Cardiac glycosides		Native of Australia	[50,51]
		Lignans	(−)-Carinol, (−)-Carissanol (−)-Nortra-chelogenin,		
		Terpenoids Alkaloids Tannins Saponins			
Pepino Fruit or sweet cucumber	*Solanum muricatum* Ait.	Phenolic acids	Hydroxy-cinnamic acid derivatives Chlorogenic acids and derivatives	Native of Peru and Chile, but widely grown in South and Central American countries and in New Zealand	[52,53]
		Pigments	β-Carotene, Chlorophyll		
Rambutan	*Nephelium lappaceum* L.	Polyphenolic compounds	Geraniin Corilagin Gallic acids Ellagic acid Ellagitannins	Native of Indonesian but widely grown in Southeast Asia	[54]
Durian	*Durio zibethinus* L.	Polyphenols Flavonoids Flavanols Anthocyanins Vitamin C Carotenoids		Native to Malaysia and Indonesia. Grown in Thailand, Indian and other South East Asian countries	[55,56]
Kiwano or horned melon	*Cucumis metuliferus* E.Mey.	Triterpenoids Alkaloids Lutein myristol, palmitol and dipalmitol phenylpropanoids, flavonoids and terpenoids		Native of south and central Africa	[57]
Kumquats (or cumquat)	*Citrus japonica* Thunb.	Essential oils Volatile compounds Limonene Germacrene D		Native to South Asia and Asia-Pacific region.	[58]
Madroño	*Garcinia madruno* (Kunth) Hammel.	Phenolic hydroxyl Groups β-Diketone bioflavonoids Polyisoprenylated benzophenones		Native to Central and South America	[59]
Prickly pear	*Opuntia ficusindica* L. Mill.	Betalain Phenolic compounds		Native of the New world, grown widely in Mexico, South Africa, Southern and Central America, Egypt, Tunisia, Algeria, Morocco, Turkey, Spain and Greece	[60]
		Flavonoids	Isorhamnetin Quercetin Kaempferol		
		Glycosides Piscidic acid			
Cupuaçu	*Theobroma grandiflorum* (Wild. ex Spring) Schumann	Dietary fibre Polyphenols Flavonoids Methyl-xanthines Proanthocyanidins Vitamin C		Native to South America countries, Colombia, Bolivia, Brazil, Pará, Peru	[61]
Miracle Fruit	*Synsepalum dulcificum* (Schumach. & Thonn.) Daniell	Epicatechin Lutein α-Tocopherol Saponin Flavonoids Tannin Alkaloids Cyanogenic glycosides	Kaempferol	Native of West Africa	[62,63]
Starfruit	*Averrhoa carambola* L.	Vitamin C Polyphenolics Flavonoids Carotenoids		Native to Asia, widely cultivated in Malaysia, Indonesia, Singapore and Hong Kong	[64,65]
Dragon fruit or pitaya fruit	*Hylocereus undatus* (Haworth) Britton & Rose	Phytosterols		Native to Central America but widely grown in Southeast Asia	[66,67]
		Betacyanins	Betanin Isobetanin Phyllocactin Hylocerenin		
		Acetic acid Polyphenols Flavonoids			
Feijoa or the pineapple guava or guavasteen	*Acca sellowiana* (O. Berg) Burret	Polyphenols Carotenoids Fatty acids		Native to South America. Also cultivated in New Zealand	[68]
Jaboticaba	*Myrciaria cauliflora* (Mart.) O.Berg or *Plinia cauliflora* (Mart.) Kausel (Branca, Sabara, Paulista, rajada var.)	Anthocyanins Polyphenols		Native to South-eastern Brazil	[69]
Araçá-pera	*Psidium acutangulum* DC.	Trihydroxy-cinnamic acid glucopyranosyl Tannin digalloyl glucopyranosyl Triterpenoid acids Vitamin C		Native of Brazilian Amazon region	[70]
Langsat	*Lansium domesticum* and *Lansium parasiticum* (Osbeck) Sahni & Bennet	Polyephenols		Native to South East Asia, widely grown in Malaysia, Thailand and Indonesia	[71]
		Onoceranoid-type triterpenoids	Lamesticumin A LamesticuminsLAnsic acid 3-ethyl ester Ethyl-lansiolate		
Longan or dragon’s eye	*Dimocarpus longan* Lour.	Phenolic acids	Ellagic acid 4-O-methyl-Gallic acid.	Native of Myanmar and Southern China, widely grown in Thailand, Cambodia and Vietnam	[72]
		Flavonoids	Quercetin glycosides, Kaempferol glycosides		
		Ellagitannin	Corilagin		
Mora de Castilla	*Rubus glaucus* Benth.	Anthocyanins Phenolic acids Ellagitannins	Sanguiin H-6 Lambertianin C	Native of Latin and South America	[73,74]
Snake fruit	*Salacca zalacca* (Gaertn.) Voss	Phenolics Flavonoids Tannins Monoterpenoids		Native to Indonesia (Java and Sumatra)	[75]
Buddha’s hand or fingered citron	*Citrus medica* L. var. sarcodactylis	Phenolic Acids Flavonones		Native of India. Cultivated and popular in China, Korea, Vietnam	[76]
		Terpenoids	Iso-limonene Citral limonene linalool, decanal nonanal		
		Vitamin C Pectin			
Soursop or graviola	*Annona muricata* L.	Acetogenins		Native of tropical forests in America, but widely grown in Southeast Asia and Asia Pacific regions	[77]
White sapote	*Casimiroa edulis* Llave	Phenolic acids Flavonoids Tannins		Native of central Mexico, but widely grown in El Salvador, Guatemala, Costa Rica, Bahamas, South Africa New Zealand, West Indies and India	[78]
Wolfberry fruit	*Symphoricarpos occidentalis* Hook.	Phenolic acids Flavonoids Carotenoids		Native of South China	[79]

**Table 3:** Bioactive compounds from vegetables processing wastes and by-products.

Vegetable	Type of Waste	Bioactive Compounds			Bioactivity	Reference
		Class	Concentration (mg/kg *)	Major Compounds		
Potato	Pulp and peel	Carbohydrate	n.a. **	Pectin	Dietary fibre, anti-obesity, hypo-cholesterolemic.	[106]
	Peel	Phenolic acids	1839–9130	Chlorogenic acid Caffeic acid	Antioxidant, anti-microbial, Anti-inflammatory.	[107,108]
		Glycoalkaloid	639–3580	α-Chaconine α-Solanine	Anti-carcinogenic (induced apoptosis in cancer cells)	
	Peel	Carotenoids	205.6	β-Carotene α-Carotene Lycopene Lutein	Antioxidant, prevention of age-related macular eye disease, pro-vitamin A.	[109,110]
Carrot	Discarded carrots	Carotenoids	1384	β-Carotene α-Carotene Lutein		
		Tocopherol	71	γ-Tocopherol		
		Carbohydrate	n.a.	Pectin	Dietary fibre Anti-obesity Hypo-cholesterolemic	
Beetroot	Pomace	Phenolic acids	1513	Ferulic acid Vanillic acid Caffeic acid Protocatechuic acid *p*-Hydroxy-benzoic acid	Antioxidant, hepatoprotective activity.	[111]
		Flavonoids	386	Catechin epicatechin, rutin		
		Betalains	558.8	Betacyanins (betanin and isobetanin) Betaxanthins (vulgaxanthin I)		
	Aerial parts (stems and leaves)	Phenolic compounds	99 mg GAE/g ***	(not identified)	Antioxidant.	[112]
Broccoli	Industrial residues: stalks and florets	Phenolic acids	74.6 (Stalks) 193.8 (Florets)	Chlorogenic acid Neochlorogenic acid Sinapic acid	Antioxidant, prevention of cancer, cardiovascular disease, and other age-related diseases.	[113]
		Flavonoids	n.d. (Stalks) 56.6 (Florets)	Kaempferol Quercetin		
		Glucosinolates	1836.6 (Stalks) 5775.6 (Florets)	Glucoiberin Glucoerucin Glucoraphanin Gluconapin Glucoalyssin Glucobrassicin Neoglucobrassin		
	Agricultural waste: leaves	Glucosinolates	1332–1594	Glucoiberin Glucoraphanin Gluconasturtiin Glucobrassicin 4-Methoxy-glucobrassicin Neoglucobrassin	Chemo-preventive effect.	[114]
Cauliflower	Stems and leaves	Phenolic acids	n.a.	Ferulic acid Sinapic acid	Antioxidant, anti-hypertensive, anti-obesity.	[115]
		Flavonoids	n.a.	Kaempferol Quercetin glycosides		
		Isothiocyanate	n.a.		Chemo-preventive	[116]
		Proteins	n.a.	Bioactive peptides from protein hydrolysate	Anti-hypertensive (ACE inhibition).	[117]

* Values are expressed as mg/kg of dry weight. ** n.a. concentration data not available in literature. *** Total phenolic content expressed as mg Gallic Acid Equivalent/g of extract.

**Table 4:** Bioactive compounds from animal and marine products waste.

Industry	Type of Waste	Bioactive Compounds	Bioactivity	Reference
Dairy products	Whey	Bioactive peptides from protein hydrolysate	Antioxidant, ACE inhibitor	[157]
		Bioactive milk oligosaccharides Neutral oligosaccharides Acidic sialylated oligosaccharides	Bifidogenic, anti-inflammatory, adherence inhibition of enteric pathogens.	[158]
		Galactooligosaccharides	Prebiotic.	[159]
	Colostrum	Lactoferrin	Antioxidant, anti-inflammatory, anti-microbial, neuroprotective.	[160]
		Oligosaccharides	Prebiotic (bifidogenic), anti-inflammatory.	[161]
Meat products	Blood: Hemoglobin Plasma	Bioactive peptides from protein hydrolysate	Opioid, Antimicrobial, ACE inhibitor.	[162]
	Trimmings and cuttings	Bioactive peptides from protein hydrolysate	Antioxidant, ACE inhibitor.	[163]
	Bones Horns Skin	Collagen hydrolysate	Beneficial effect on bone metabolism, Antioxidant, ACE inhibitor.	
Marine products	Shrimp shells, heads and tails	Chito-oligosaccharides	Antioxidant.	[164]
		Astaxanthin	Antioxidant, anti-cancer, neuroprotective, anti-aging.	[165]
		Polyunsaturated fatty acids Ω3	Beneficial effects on cardiovascular disease, autoimmune diseases and mental health disorders.	[166]
	Salmon nasal cartilage	Proteoglycans	Anti-angiogenesis, relieving joint pain discomfort, promote wound healing.	[167,168,169]
	Salmon skin and trimmings	Bioactive peptides from protein hydrolysate	Anti-diabetic, antioxidant, ACE inhibitor, enhancing learning and memory in aged mice	[170,171,172]
